# Optimization of PCR conditions for amplifying an AT-rich amino acid transporter promoter sequence with high number of tandem repeats from *Arabidopsis thaliana*

**DOI:** 10.1186/s13104-017-2982-1

**Published:** 2017-11-28

**Authors:** Pinky Dhatterwal, Sandhya Mehrotra, Rajesh Mehrotra

**Affiliations:** Department of Biological Sciences, Birla Institute of Technology & Sciences, Pilani, Rajasthan 333031 India

**Keywords:** AT-rich, PCR, Optimization, Tandem repeats, Extension

## Abstract

**Objective:**

The aim of the present study is to optimize the PCR conditions required to amplify the promoter sequence of an amino acid transporter having an AT-rich base composition with a high number of tandem repeats.

**Result:**

Results show that successful amplification can be achieved by performing a 2-step PCR at a lower extension temperature of 65 °C for an increased extension period of 1.5 min/kb, with MgCl_2_ concentration ranging from 2.5 to 3.0 mM. The results also suggest that the DNA concentration of about 25–30 ng/µl was essential to achieve this amplification.

## Introduction

PCR is one of the indispensable techniques in molecular biology for in vitro amplification of a specific segment of DNA [1]. It is highly reliable because of its sensitivity, accuracy, and speed [2, 3]. However, under specific requirements such as to amplify templates, which are AT- or GC-rich, or have a high number of tandem repeats the PCR conditions need to be optimized. Plant promoter regions are generally difficult to amplify by PCR as they are highly AT-rich and sometimes contain tandem repetitive DNA sequences [4, 5]. Tandem repeats represent two or more copies of short segments of DNA occurring repeatedly from head-to-tail within the coding and regulatory regions [6]. The problem with these templates is that they need lower annealing and extension temperatures which can result in the amplification of undesired products [7, 8]. The aim of this work is to amplify a promoter sequence (1781 bp) of an amino acid transporter (AT2G40420) from *Arabidopsis thaliana*, which is highly AT-rich and has a high number of tandem repeats.

**Fig. 1:**
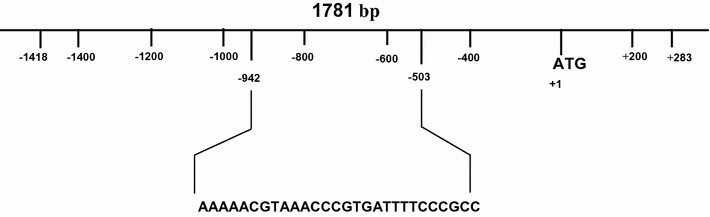
Amino acid transporter (AT2G40420) promoter region (1781 bp). The sequence and position of 28 bp long tandem repeat, occurring 15.5 times in the promoter region from − 503 to − 942 and the translation start site ATG, are depicted in the figure

The in silico analysis of the promoter sequence reveals that it possesses many important *cis*-acting regulatory elements such as light-responsive, auxin-responsive, salicylic acid-responsive, and abscisic acid-responsive elements along with 16 copies of an ACGT motif [9]. Studies suggest that the *cis*-elements with ACGT core sequence responds to light, anaerobiosis, phytohormones like abscisic acid, jasmonic acid, salicylic acid and auxin [10, 11]. Furthermore, Zou et al. [12] conducted a study where they concluded that around 19.6% of the total pCREs (putative *cis*-regulatory elements) identified in the promoter regions of abiotic stress responsive genes have ACGT as a core sequence. Therefore, tapping this promoter sequence for its response to abiotic stress conditions can potentially bring forth important characteristics that can further find wide application for generation of transgenic plants with high stress tolerance. As, a suitable promoter is needed to achieve desired expression levels of a transgene [13]. In the study, the promoter sequence (AT2G40420, 1781 bp) was amplified from *Arabidopsis thaliana* genome. However, the sequence is 65.2% AT-rich and has 15.5 copies of 28 base long tandem repeat [14], which makes it difficult to amplify by PCR (Fig. 1). These tandem repeat sequences have a binding site for bZIP (basic leucine zipper) transcription factors (TFs). Reports suggest that tandem repeats possessing binding sites for transcription factors in the promoter regions can affect the transcriptional rate of a gene [15]. To check the effect of all these TF binding sites localized in tandem repeats on the downstream gene expression, isolation of the promoter sequence with all the copies of tandem repeats was highly desirable.

## Main text

### Methods

#### Plant material and growth conditions

*Arabidopsis thaliana*, ecotype Columbia (Col-0) was used in this study. *Arabidopsis* seeds were procured from LEHLE SEEDS Company (Catalog number: WT-02), Texas, USA. Seeds were vernalized at 4 °C in the dark for 3 days before sowing in 5.08 cm plastic pots filled with soilrite. Thereafter, the pots were transferred into a growth chamber (Daihan Labtech, LGC-5101, India) maintaining a 16-h light/8-h dark photoperiod, 22 °C temperature, 75% relative humidity. After 3 weeks, rosette leaves were harvested to extract the genomic DNA.

#### Genomic DNA isolation

Genomic DNA was isolated from the leaves of *Arabidopsis thaliana* (ecotype Col-0) using the DNeasy Plant Mini Kit (Qiagen, Cat No./ID: 69104) according to the manufacturer’s recommendations. The DNA integrity was confirmed by running 0.8% agarose gel electrophoresis at 80 V for 30 min.

#### Primer design

Primers were designed to amplify a 1781 bp promoter sequence of the amino acid transporter (AT2G40420) (Table 1) using the Primer3 program [16]. Their specificity was ensured by performing primer-BLAST (https://www.ncbi.nlm.nih.gov/tools/primer-blast/) with the *Arabidopsis* genome. Further, the OligoAnalyzer tool supported by Integrated DNA Technologies (https://www.idtdna.com/SciTools/SciTools.aspx.) was used to check for the presence of any secondary structure or primer–dimer formation.

**Table 1:** Forward and reverse primer specifications for AT2G40420 promoter sequence

Primer	Primer sequence (5′→3′)	Tm °C	GC%	Product size
AT2G40420F	CCTACTAGTTCGTGATACTG	52.05	45.00	1781 bp
AT2G40420R	CGAACGATTCCTTCATCACG	57.02	50.00	

#### PCR conditions

Each 20 µl PCR contained 2 μl of genomic DNA ( ~ 50 ng), 4 µl of 5X Phusion HF buffer, 0.4 µl of 10 mM dNTPs, 0.8 µl of each 10 µM forward and reverse primer, 0.2 µl of Phusion DNA polymerase (2U/µl), and varying concentrations of MgCl_2_ ranging from 1.5 to 3.5 mM. All the reagents were procured from Thermo Fisher SCIENTIFIC (Catalog number: F530S, Waltham, MA, USA) and MB grade nuclease-free water from Himedia (Catalog number: ML024). A 2-step PCR was carried out using the Applied Biosystems^®^ Veriti^®^ 96-Well Thermal Cycler (Catalog number: 4375786, Foster City, CA, USA) with conditions as follows: Initial denaturation at 98 °C for 1.5 min; followed by 35 cycles of denaturation at 98 °C for 30 s, extension at 60/65/68/72 °C for 3 min and final extension at 60/65/68/72 °C for 7 min. PCR for each extension temperature with varying MgCl_2_ concentrations were performed separately and in triplicates. PCR products were checked by electrophoresis in 1% (w/v) agarose gel, at 80 V for 30 min.

#### Amplicon sequence analyses

The QIAquick Gel Extraction Kit (Qiagen, Catalog number: 28704) was used to purify the PCR products. The purified PCR product along with the primers used for amplification, was then directed for sequencing to verify the specificity of the amplified product. The amplicon specificity was confirmed by analysing the obtained sequencing results with the reference sequence deposited in the TAIR database (https://www.arabidopsis.org) of the amino acid transporter promoter region [17].

## Results and discussion

### Concentration of magnesium ions

**Fig. 2:**
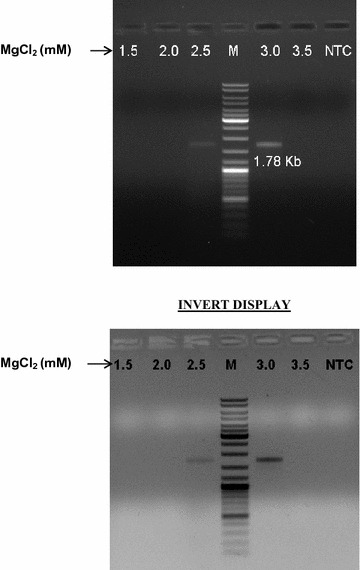
Effects of MgCl_2_ concentration on PCR amplification at an extension temperature of 65 °C. Lane M: 10 kb DNA ladder; lane 1: 1.5 mM MgCl_2_; lane 2: 2 mM MgCl_2_; lane 3: 2.5 mM MgCl_2_; lane 5: 3 mM MgCl_2_; lane 6: 3.5 mM MgCl_2_; lane 7: no-template negative control

The magnesium ion concentration greatly influences the PCR as DNA polymerase requires Mg^2+^ ions for its proper functioning [18, 19]. Therefore, to achieve maximal PCR yield the MgCl_2_ concentration needs to be optimized. As, a high Mg^2+^ ion concentration can hinder the reaction by preventing proper melting of template DNA and can also promote non-specific binding of primers. Even a low Mg^2+^ ion concentration can adversely affect the product yield. With this aim, varying concentrations of MgCl_2_ such as 1.5, 2.0, 2.5, 3.0, 3.5 mM were tried. The desired amplicon yield was obtained at a 3.0 mM MgCl_2_ concentration (Fig. 2).

### Extension temperature

For successful amplification, the extension time and temperature need to be carefully optimized. Xin-Zhuan Su et al. [20] reported that to amplify an AT-rich DNA, reduced extension temperatures are needed. In the present study, a two-step PCR (denaturation and amplification) was performed at four different extension temperatures 60, 65, 68 and 72 °C with increased extension time from the usual 1 to 1.5 min/kb. Successful amplification was achieved at an extension temperature of 65 °C with 2.5 mM MgCl_2_ yielding a faint band while an intense band was observed with 3 mM MgCl_2_ concentration (Fig. 2). No results were obtained at other extension temperatures (60, 68, and 72 °C) at any of the five MgCl_2_ concentrations tested (data not shown).

## Conclusions

The promoter region of the amino acid transporter was difficult to amplify by PCR owing to its high AT content and a high number of tandem repeats. Successful amplification can be achieved, after optimization of MgCl_2_ concentration and extension temperature with the DNA template of desired concentration.

## Limitations

- The DNA template should be pure, homogeneous and concentration should be around 50–60 ng for setting up a 20 µl PCR reaction.
- Efficient for AT-rich DNA templates.

## Abbreviations

dNTP: deoxyribonucleotide triphosphate
PCR: polymerase chain reaction
TRs: tandem repeats
TFs: transcription factors
pCREs: putative *cis*-regulatory elements

## References

[1] Garibyan L, Avashia N (2013). Polymerase chain reaction. J Invest Dermatol.

[2] Coleman WB, Tsongalis GJ, Coleman WB, Tsongalis GJ (2006). The polymerase chain reaction. Molecular diagnostics for the clinical laboratorian.

[3] Obradovic J, Jurisic V, Tosic N, Mrdjanovic J, Perin B, Pavlovic S (2013). Optimization of PCR conditions for amplification of GC-rich EGFR promoter sequence. J Clin Lab Anal.

[4] Sahdev S, Saini S, Tiwari P, Saxena S, Saini KS (2007). Amplification of GC-rich genes by following a combination strategy of primer design, enhancers and modified PCR cycle conditions. Mol Cell Probes.

[5] Gemayel R, Cho J, Boeynaems S, Verstrepen KJ (2012). Beyond junk-variable tandem repeats as facilitators of rapid evolution of regulatory and coding sequences. Genes.

[6] Quilez J, Guilmatre A, Garg P, Highnam G, Gymrek M, Erlich Y (2016). Polymorphic tandem repeats within gene promoters act as modifiers of gene expression and DNA methylation in humans. Nucleic Acids Res.

[7] Hommelsheim CM, Frantzeskakis L, Huang M, Ülker B (2014). PCR amplification of repetitive DNA: a limitation to genome editing technologies and many other applications. Sci Rep.

[8] Kennedy S, Oswald N (2011). PCR troubleshooting and optimization: the essential guide.

[9] Lescot M, Déhais P, Thijs G, Marchal K, Moreau Y, Van de Peer Y, Rouzé P, Rombauts S (2002). PlantCARE, a database of plant *cis*-acting regulatory elements and a portal to tools for in silico analysis of promoter sequences. Nucleic Acids Res.

[10] Mehrotra R, Yadav A, Bhalothia P, Karan R, Mehrotra S. Evidence for directed evolution of larger size motif in *Arabidopsis thaliana* genome. Sci World J. 2012; 1–5. 10.1100/2012/983528. 10.1100/2012/983528 PMC3354754 22645502

[11] Mehrotra R, Sethi S, Zutshi I, Bhalothia P, Mehrotra S (2013). Patterns and evolution of ACGT repeat *cis*-element landscape across four plant genomes. BMC Genom.

[12] Zou C, Sun K, Mackaluso JD, Seddon AE, Jin R, Thomashow MF, Shiu SH (2011). Cis-regulatory code of stress-responsive transcription in *Arabidopsis thaliana*. Proc Natl Acad Sci USA.

[13] Potenza C, Aleman L, Sengupta-Gopalan C (2004). Invited review: targeting transgene expression in research, agricultural, and environmental applications: promoters used in plant transformation. InVitro Cell Dev Biol Plant.

[14] Chow CN, Zheng HQ, Wu NY, Chien CH, Huang HD, Lee TY (2016). PlantPAN 2.0: an update of plant promoter analysis navigator for reconstructing transcriptional regulatory networks in plants. Nucleic Acids Res.

[15] Vinces MD, Legendre M, Caldara M, Hagihara M, Verstrepen KJ (2009). Unstable tandem repeats in promoters confer transcriptional evolvability. Science.

[16] Untergasser A, Cutcutache I, Koressaar T, Ye J, Faircloth BC, Remm M (2012). Primer3—new capabilities and interfaces. Nucleic Acids Res.

[17] Berardini TZ, Reiser L, Li D, Mezheritsky Y, Muller R, Strait E (2015). The Arabidopsis information resource: making and mining the “gold standard” annotated reference plant genome. Genesis.

[18] Ekman S (1999). PCR optimization and troubleshooting, with special reference to the amplification of ribosomal DNA in lichenized fungi. Lichenologist.

[19] Cao Y, Zheng Y, Fang B (2004). Optimization of polymerase chain reaction-amplified conditions using the uniform design method. J Chem Technol Biotechnol.

[20] Su XZ, Wu Y, Sifri CD, Wellems TE (1996). Reduced extension temperatures required for PCR amplification of extremely A+T-rich DNA. Nucleic Acids Res.

